# ESG disclosure quality and firm valuation under economic policy uncertainty: Evidence from Chinese A-share listed firms

**DOI:** 10.1371/journal.pone.0330278

**Published:** 2026-03-18

**Authors:** Chia-Hsien Tang, Feifei Luo

**Affiliations:** School of Business, Guangxi Minzu Normal University, Chongzuo, Guangxi, China; Iwate University: Iwate Daigaku, JAPAN

## Abstract

This study examines the extent to which high-quality environmental, social, and governance (ESG) disclosure mitigates the effects of economic policy uncertainty (EPU) on enterprises’ market valuation. The research indicates that utilizing panel data from Chinese A-share listed companies reveals that Economic Policy Uncertainty (EPU) significantly diminishes firm value, whereas robust Environmental, Social, and Governance (ESG) disclosure mitigates this adverse effect. Robustness checks employing alternative variable definitions and subsample analyses based on ownership type, area, and industry corroborate these findings. Mediation analysis indicates that the indirect effects, mediated through a valuation-based channel, reveal that EPU-induced reductions in business value diminish analyst coverage and exacerbate financial distress. These findings offer significant insights for corporate governance, investor decision-making, and regulatory frameworks in emerging nations, as they demonstrate the strategic importance of ESG disclosure in bolstering business resilience against heightened policy risk.

## 1 Introduction

Recent business strategies increasingly emphasize environmental, social, and governance (ESG) considerations, reflecting their growing importance to both academics and investors. Rather than being viewed solely as regulatory obligations, ESG disclosures are now widely regarded as strategic tools that can enhance firm value by reducing information asymmetry, strengthening stakeholder trust, and improving corporate reputation [1–3]. This shift mirrors a broader transformation in global capital markets, where sustainability-related practices are progressively incorporated into investment decisions and firm valuation models [4].

China has closely aligned with this global trend through a series of ESG-oriented policy initiatives. These include regulatory guidance issued by the China Securities Regulatory Commission (CSRC) and major stock exchanges, as well as national commitments to peak carbon emissions by 2030 and achieve carbon neutrality by 2060. In particular, recent regulatory guidance issued by the China Securities Regulatory Commission (CSRC) and China’s major stock exchanges emphasizes 2024 as a critical transition year for the standardization of ESG disclosure practices, with more comprehensive and mandatory sustainability reporting requirements scheduled to be progressively implemented by 2026, with comprehensive ESG reporting expected to become mandatory by 2026 [5, 6]. However, the effectiveness of these initiatives depends not only on regulatory compliance, but also on whether ESG disclosures can meaningfully influence firm outcomes especially corporate valuation under volatile macroeconomic conditions such as heightened economic policy uncertainty (EPU).

Economic policy uncertainty has risen sharply in recent years, driven by geopolitical tensions, trade disputes, pandemic-related shocks, and inflationary pressures [7]. In the Chinese context, events such as the U.S.–China trade conflict and the COVID-19 pandemic have highlighted the disruptive effects of policy uncertainty on market confidence and investor behavior [8]. Prior studies show that elevated EPU tends to suppress corporate investment, increase financing costs, and depress firm value by intensifying investor risk aversion [9]. In such an environment, transparent and credible ESG disclosures may serve as signals of long-term strategic stability and organizational resilience, thereby sustaining investor confidence and partially shielding firms from valuation losses [2, 4].

Although the literatures on ESG disclosure and economic policy uncertainty have expanded rapidly, empirical evidence on their combined effects remains limited, particularly in developing economies. Existing studies suggest that greater ESG transparency can alleviate financing constraints and enhance firm value [4,10], yet they rarely examine whether these benefits vary with the macroeconomic environment. In particular, little attention has been paid to the moderating role of ESG disclosure under conditions of heightened policy uncertainty. Moreover, most prior research focuses on developed markets, offering limited insights into institutional settings such as China, where state ownership, policy-driven governance, and regulatory volatility may shape how ESG signals are interpreted by investors [11]. In addition, the downstream consequences of EPU-induced valuation shocks such as changes in analyst coverage and financial distress remain underexplored, despite their importance for understanding the broader financial implications of policy risk.

The study uses a large panel dataset of Chinese A-share listed companies to investigate whether the quality of ESG disclosure moderates the relationship between economic policy uncertainty and business valuation, therefore filling in some of these gaps. Combining information asymmetry theory and signaling theory, we propose ESG disclosure as an instrument of expressing internal resilience and long-term orientation by companies, hence lowering uncertainty for investors. Furthermore anchored in stakeholder theory, we argue that companies with more ESG openness are more in line with stakeholder expectations, which helps them to resist valuation pressure in times of macroeconomic turbulence.

This study makes three main contributions to the literature. First, it contributes to the literature on economic policy uncertainty and firm valuation by providing evidence that the valuation impact of policy uncertainty is conditional on firms’ ESG disclosure quality. By focusing on emerging markets, the study highlights ESG disclosure as a risk-mitigating informational mechanism that becomes particularly relevant when policy uncertainty intensifies. Second, the study advances the ESG and corporate finance literature by examining the downstream economic consequences of policy uncertainty through a mediation framework. By documenting how EPU-induced valuation changes affect analyst coverage and financial distress, the paper extends existing research beyond contemporaneous valuation effects to the broader information environment and financial stability of firms. Third, the study offers new insights into the role of ESG disclosure by distinguishing disclosure quality from ESG performance and by emphasizing its state-dependent value under persistent, policy-driven uncertainty. Unlike prior studies that focus on short-term crisis episodes (e.g., [12, 2]), this paper shows that ESG disclosure functions as a conditional signaling mechanism in a sustained uncertainty context, thereby clarifying its distinct role in shaping firm valuation.

Our results reveal several key insights. Higher levels of economic policy uncertainty are consistently associated with lower firm valuation, confirming its adverse effect on market expectations. However, this negative impact is significantly attenuated for firms with higher-quality ESG disclosures. The buffering effect is particularly pronounced among private firms, companies located in economically developed eastern regions, and non-manufacturing sectors. Further analysis shows that changes in firm valuation statistically mediate the impact of EPU on analyst coverage and financial distress, highlighting the broader informational and financial consequences of policy uncertainty.

The paper is structured as follows: Sect 2 reviews the relevant literature. Sect 3 details the methodology, data, and model. Sect 4 presents the empirical results. Sect 5 concludes with a discussion of the findings, limitations, and avenues for future research.

## 2 Literature and hypothesis development

### ESG disclosure quality and firm value

Environmental, Social, and Governance (ESG) disclosure quality has attracted growing attention in the corporate finance literature as an important determinant of firm value. From the perspective of information asymmetry, high-quality ESG disclosure enhances transparency regarding firms’ governance structures, risk management practices, and long-term strategic orientation, thereby reducing informational gaps between firms and investors [13]. Improved transparency allows investors to more accurately assess firm fundamentals, which can translate into higher market valuation.

Signaling theory further suggests that ESG disclosure can function as a credible signal of firm quality. Producing comprehensive and consistent ESG disclosures requires organizational commitment and nontrivial resources. Firms that voluntarily provide high-quality ESG information are therefore more likely to possess stronger governance mechanisms and a long-term orientation, characteristics that investors may reward in valuation. This signaling role of ESG disclosure is particularly relevant in capital markets where reputation and credibility play an important role in access to external financing.

Empirical evidence from China supports this view [14]. As the Chinese market moves toward more standardized ESG disclosure requirements, recent studies show that higher-quality ESG disclosure alleviates financing constraints and enhances firm valuation [4,10]. These findings suggest that ESG disclosure quality is becoming increasingly relevant in capital allocation decisions within China’s evolving institutional environment.

At the same time, the relationship between ESG disclosure and firm value remains debated. Some studies argue that ESG-related activities and disclosures may impose short-term costs, such as increased compliance burdens or misallocation of resources, which could offset long-term benefits [15]. These concerns are particularly salient in emerging markets like China, where ESG standards are still developing and disclosure practices remain heterogeneous across firms and regions. Given these mixed findings, establishing the baseline relationship between ESG disclosure quality and firm valuation in the Chinese context is a necessary first step.

Accordingly, we propose the following hypothesis

Hypothesis 1: ESG disclosure quality is positively associated with firm value among Chinese A-share listed companies.

### Economic policy uncertainty and firm valuation

Economic policy uncertainty (EPU) has emerged as a major source of macroeconomic risk influencing firm behavior and market valuation [16]. Elevated policy uncertainty increases ambiguity regarding future regulatory conditions, taxation, and government intervention, making it more difficult for investors to form expectations about firms’ future cash flows and discount rates. As a result, investors tend to demand higher risk premia and adopt more conservative valuation assessments [17,18,19].

China, where continuous policy changes, major government participation, and regulatory opacity create an environment of enormous institutional uncertainty, clearly shows these effects. Research specifically tailored for China confirm that EPU has a quite negative effect on company performance. Rising policy uncertainty, for example, degrades investment efficiency and decreases corporate performance across several sectors and ownership structures Chen et al. (2023) [20] and Meng et al. [21] document. Reflect preventative behavior in the face of policy uncertainty by finding that Chinese enterprises react to EPU by keeping more cash and cutting capital expenditures.

From an information asymmetry perspective, heightened policy uncertainty amplifies informational frictions between firms and investors. When policy signals become less predictable, external information becomes less informative, and uncertainty surrounding firms’ operating environments increases. This mechanism is particularly relevant in China, where frequent policy adjustments and substantial government involvement intensify uncertainty about regulatory direction and firm prospects.

Empirical studies focusing on Chinese firms consistently document the adverse valuation effects of economic policy uncertainty [22]. Rising EPU has been shown to reduce investment efficiency, increase precautionary cash holdings, and depress firm performance across industries and ownership structures [21,20]. These findings highlight the importance of establishing the baseline relationship between EPU and firm valuation within China’s institutional setting. Based on this reasoning, we propose the following hypothesis:

Hypothesis 2: Economic policy uncertainty is negatively associated with firm value among Chinese A-share listed companies.

### The moderating influence of ESG disclosure on the relationship between economic policy uncertainty and firm value

While the negative impact of economic policy uncertainty on firm valuation is well documented, less is known about whether firm-level disclosure practices can mitigate this effect. Signaling theory suggests that when external policy signals become less reliable, investors increasingly rely on firm-specific information to assess long-term prospects. In such environments, high-quality ESG disclosure may serve as a stabilizing signal [23,24] by conveying credible information about firms’ governance quality, risk management capacity, and commitment to sustainable strategies.

Under heightened policy uncertainty, ESG disclosure can therefore reduce information asymmetry and reassure investors about firms’ resilience, making firm valuation less sensitive to adverse macroeconomic shocks. This view is consistent with the notion of an “insurance effect,” whereby credible disclosure helps preserve investor confidence during periods of uncertainty.

At the same time, existing empirical evidence on this moderating relationship remains limited and largely concentrated in developed markets or crisis-specific contexts. Studies such as Broadstock et al. [2] and Bannier et al. [12] focus primarily on short-term systemic crises [25], leaving open the question of whether ESG disclosure plays a similar buffering role under persistent, policy-driven uncertainty in emerging markets like China.

To address this gap, we examine the interaction between ESG disclosure quality and economic policy uncertainty in shaping firm valuation. We propose the following hypothesis:

Hypothesis 3 ESG disclosure quality attenuates the negative effect of economic policy uncertainty on firm value.

### Economic consequences: Analyst attention and financial distress

Beyond contemporaneous valuation effects, economic policy uncertainty may have broader consequences for firms’ information environment and financial stability. Declines in firm value can reduce analyst coverage, as analysts tend to allocate attention toward firms perceived as more valuable and informative. Reduced analyst following weakens external monitoring and exacerbates information asymmetry, further impairing market efficiency [6] (Zhang and Liu 2021).

Lower firm valuation is also closely linked to financial vulnerability. Firms experiencing valuation declines may face deteriorating creditworthiness, constrained access to external financing, and increased exposure to liquidity shocks. These risks are particularly pronounced in emerging markets [26], where institutional protections and financial safety nets are relatively limited [27]. As valuation weakens, firms become more susceptible to financial distress, threatening long-term viability [28, 29].

Building on this logic, we examine whether firm value serves as a transmission channel through which economic policy uncertainty affects analyst attention and financial distress. Accordingly, we propose the following hypothesis:

Hypothesis 4: Economic policy uncertainty affects analyst coverage and financial distress through its impact on firm value.

## 3 Methods

### Sample selection and data sources

This study investigates the relationship among ESG disclosure quality, economic policy uncertainty (EPU), and business valuation employing a panel dataset of Chinese A-share listed companies spanning the period from 2011 to 2023. Adapted from the China Stock Market (CSMAR) database are firm-level financial and governance statistics comprising total assets, liabilities, profitability measures, and ownership structure. Adaptable from the Wind Financial Terminal, the Huazheng ESG Rating System generates ESG ratings. The China EPU Index created by Baker, Bloom, and Davis [ 30,31] provides an indicator of economic policy uncertainty that has been widely utilized in studies on macroeconomic risk.

The sample was modified using the following steps to guarantee consistency and quality of data: The sample selection process follows several steps. First, firms classified as “ST” or “*ST” were excluded due to financial distress or abnormal operations. Second, financial institutions, including banks, insurance companies, and securities firms, were removed because of their distinct regulatory environment and accounting standards. Third, firms that conducted initial public offerings (IPOs) during the sample period were excluded to avoid distortions from incomplete financial histories. Finally, observations with missing ESG ratings or key financial variables were dropped. All continuous variables were winsorized at the 1st and 99th percentiles to mitigate the influence of extreme outliers, a common practice in empirical corporate finance studies. Descriptive statistics before and after winsorization indicate that this procedure substantially reduces the impact of extreme values without altering the central tendency of the variables. These filters produce a final balanced panel dataset with 34,607 firm-year observations.

To ensure data consistency, firms with missing ESG disclosure information were excluded from the sample. We further exclude firm-year observations with missing values for key financial variables required to construct Tobin’s Q and control variables. Firms that were delisted during the sample period are retained up to their last available fiscal year, following standard practice in the panel-data literature.

### Variables definition

#### Dependent variable.

Business market value, which is the ratio of market value to book value of assets (excluding goodwill and intangible assets) and is proxied by Tobin’s Q, is the main outcome variable for business valuation. Widely accepted as a forward-looking assessment of company performance and valuation, Tobin’s Q.

Operating on the China-specific index created by Baker et al. [30], which indicates the frequency of newspaper-based allusions to economic policy-related uncertainty. Key Independent Variable of Economic Policy Uncertainty (EPU) is operationalized. Annually averaged, the index provides a macro-level approximation of policy-induced uncertainty.

#### Key independent variable.

Economic Policy Uncertainty (EPU) is operationalized using the China-specific index developed by Baker et al. [30], which reflects the frequency of newspaper-based references to economic policy-related uncertainty. The index is averaged annually and serves as a macro-level proxy for policy-induced uncertainty. During most of the sample period, ESG disclosure in China is best characterized as semi-voluntary. While stock exchanges and regulators have issued guidance and encouraged ESG reporting, firms retain substantial discretion regarding the scope, depth, and consistency of disclosure. This institutional setting leads to meaningful cross-firm variation in ESG disclosure quality, even under a shared regulatory framework.

#### Moderating variable.

ESG disclosure quality is measured using the Huazheng ESG Rating Index obtained from the Wind Financial Terminal. According to the official Huazheng methodology, the rating system evaluates firms along three first-tier pillars Environmental (E), Social (S), and Governance (G) which are further decomposed into multiple thematic dimensions and key indicators. Specifically, the framework covers 3 pillars, 14 second-tier themes, and 26 core indicators, supported by a broader set of underlying data points, with industry-specific weighting schemes applied during aggregation (Huazheng ESG Rating Methodology, Wind).

Huazheng assigns firms to nine ordered rating grades AAA, AA, A, BBB, BB, B, CCC, CC, and C from highest to lowest ESG disclosure quality. Following common practice in prior empirical studies using the Wind–Huazheng ESG ratings, we convert these ordered categories into a numerical score ranging from 9 to 1 (AAA = 9, …, C = 1) to facilitate regression estimation and interaction analysis.

Although the Huazheng ESG index is commonly labeled as an ESG rating, it is primarily constructed based on firms’ publicly disclosed ESG-related information, including annual reports, sustainability reports, and regulatory filings. In the Chinese institutional context, where outcome-based ESG metrics and third-party verification remain limited, ESG ratings largely reflect the quality, completeness, and consistency of ESG disclosure rather than realized ESG performance.

Following prior studies using Chinese ESG data [4, 32], we therefore interpret the Huazheng ESG rating as a disclosure-based proxy and use it to measure ESG disclosure quality in our empirical analysis. This coding approach allows us to preserve the ordinal information embedded in the rating system while facilitating interaction analysis and comparability with prior empirical studies.

To ensure that our results are not driven by this coding choice, we conduct robustness checks using alternative ESG specifications, including an alternative continuous Huazheng ESG score where available. The main results remain qualitatively unchanged, suggesting that our findings are not sensitive to the specific operationalization of the ESG variable, including an alternative Huazheng ESG numerical score where available, and the main findings remain qualitatively unchanged.

#### Control variables.

We consider firm-level heterogeneity that could hide the correlation between ESG disclosure, economic policy uncertainty, and company valuation by means of a broad spectrum of control variables grounded on literature [3,20].

Leverage (Lev), the measure of liabilities to assets, reveals financial risk and capital structure. ROA, net income divided by average total assets measures profitability and operational efficiency. Mature and market experience are indicated by Firm Age, the natural logarithm of one plus the years since an IPO. By use of a comparison between fixed assets and total assets, the Fixed Asset Ratio (Ppe) addresses asset tangibility and capital intensity. Comparatively to operating income, cash flow from running operations shows internal financing capability and liquidity. Showing the percentage of shares owned by institutional investors, institutional ownership (IIR) indicates external monitoring and governance quality.

We also include audit quality using a binary variable. The Big Four Auditor (Big4) variable is one if a global Big Four accounting firm audits a company’s financial records; else, it is 0. The ratio of the second-to- fifth-biggest owners’ shareholdings to the largest shareholder is known as the ownership balance, Shb. This benchmark gauges internal governance and ownership concentration. These restrictions guarantee that important firm-level valuation components are included into our empirical models. Industry fixed effects are included into all regression models to adjust for sectoral differences and unobservable variance. Year fixed effects are omitted in the baseline specification because the EPU variable varies only over time. Including a full set of year dummies would absorb most of the variation in EPU and lead to severe multicollinearity. As reported in the robustness section, we nevertheless re-estimate all main models with year fixed effects, and the moderating effect of ESG disclosure remains robust. EPU’s time-varying characteristics help it to implicitly consider annual macroeconomic shocks. Table 1 contains all variables together with their notation, definitions, and sources of data.

**Table 1:** Variable definitions.

Variable Name	Abbreviation	Notation in Equations	Full Definition and Data Source
Firm Market Value (Dependent Variable)	TobinQ	TobinQ	Ratio of market value of equity to total assets minus net intangible assets and goodwill.. Source: Calculated from CSMAR financial data.
Economic Policy Uncertainty (Independent Variable)	EPU	EPU	Annual China Economic Policy Uncertainty Index constructed by Baker et al. [30], based on the frequency of newspaper references to policy-related uncertainty. Higher values indicate greater policy-related uncertainty. Source: Baker, Bloom & Davis EPU index (policyuncertainty.com).
ESG Disclosure Quality (Moderating Variable)	ESG	ESG	ESG disclosure quality measured by the Huazheng ESG Rating Index. The rating system classifies firms into nine categories (AAA, AA, A, BBB, BB, B, CCC, CC, C). For empirical analysis, ratings are converted into scores from 1 (C) to 9 (AAA). Higher scores indicate better ESG transparency. Source: Wind Financial Database (Huazheng ESG Rating Index).
Leverage Ratio (Financial leverage)	Lev	Lev	Total liabilities divided by total assets. Source: CSMAR.
Ownership Balance (Concentration balance)	Shb	Shb	Ratio of the shareholding of the 2nd–5th largest shareholders to that of the largest shareholder. Source: CSMAR.
Return on Assets (Profitability)	Roa	Roa	Total profit divided by average total assets (for the year). Source: CSMAR.
Firm Age	Age	Age	Natural logarithm of (current year – IPO year + 1), representing the firm’s age in years since IPO. Source: CSMAR.
Fixed Asset Ratio	Ppe	Ppe	Book value of fixed assets divided by total assets (asset tangibility). Source: CSMAR.
Operating Cash Flow	Cash	Cash	Net cash flow from operating activities divided by operating revenue. Source: CSMAR.
Managerial Ownership	Mgr	Mgr	Percentage of shares held by firm executives (or CEO) relative to total shares outstanding. Data source: CSMAR.
Institutional Ownership	IIR	IIR	Institutional investors’ shareholdings as a fraction of total shares outstanding. Source: CSMAR.
Big Four Auditor (Dummy)	Big4	Big4	Dummy variable equal to 1 if the firm’s financial statements are audited by a Big Four accounting firm, and 0 otherwise. Source: CSMAR.
Industry (Industry Fixed Effects)	Ind	Ind	Industry dummy variables to control for sector-specific factors. Source: Classified per CSRC industry categories (included in models).
Year (Year Fixed Effects)	Year	Year	Year dummy variables.
Analyst Coverage	Analyst	Analyst	Natural logarithm of (1 + number of financial analysts covering the firm). Source: CSMAR (analyst coverage data).
Financial Distress (Altman Z-score)	Z-score	Z	Altman Z-score measuring the likelihood of financial distress. Lower values indicate higher distress. Computed following Altman [33]. Source: CSMAR (calculated from financial statements).

#### Methodology.

The empirical strategy comprises three main components: (1) a baseline fixed-effects panel regression to estimate the effect of economic policy uncertainty (EPU) on firm valuation; (2) a moderation model to test whether ESG disclosure quality mitigates this effect; and (3) an extended mediation model to explore the downstream economic consequences via analyst coverage and financial distress.

We first estimate the baseline model to evaluate the direct impact of EPU on firm value, proxied by Tobin’s Q:


$${\text{TobinQ}}_{\text{i},\text{t}}=\beta_{\text{0}}+\beta_{\text{1}}{\text{EPU}}_{\text{t}}+\sum \beta_{\text{m}}{\text{CVS}}_{\text{i},\text{t}}+\sum {\text{Ind}}_{\text{i}}+\epsilon_{\text{i},\text{t}}$$ (1)

Where,$\;{\text{TobinQ}}_{\text{i},\text{t}}$ denotes the firm value for firm i in year t, ${\text{EPU}}_{\text{t}}$ is the economic policy uncertainty index, and Controls, ${\text{CVS}}_{\text{i},\text{t}}\;$includes financial and governance-related control variables (e.g., leverage, profitability, firm age). Industry fixed effects are included to account for sector-specific heterogeneity. Standard errors are clustered at the firm level.

Industry fixed effects are included in all baseline regressions to control for time-invariant sectoral heterogeneity. Year fixed effects are not included in the baseline specification because the Economic Policy Uncertainty (EPU) index is a purely time-series macroeconomic variable. Including a full set of year dummies would absorb most of the variation in EPU, leading to severe multicollinearity and mechanically attenuating the estimated coefficient on EPU.

To address potential concerns regarding unobserved time-varying macroeconomic shocks, we re-estimate all baseline and moderation models with year fixed effects as part of robustness analyses and report the results separately.

To explore the moderating effect of ESG disclosure quality, we incorporate an interaction term between EPU and ESG:


$${\text{TobinQ}}_{\text{i},\text{t}}=\alpha_{\text{0}}+\alpha_{\text{1}}{\text{EPU}}_{\text{t}}+\alpha_{\text{2}}{\text{ESG}}_{\text{i},\text{t}}+\alpha_{\text{3}}{\text{EPU}}_{\text{t}}\times {\text{ESG}}_{\text{i},\text{t}}+\sum \alpha_{\text{m}}{\text{CVS}}_{\text{i},\text{t}}+\sum {\text{Ind}}_{\text{i}}+\epsilon_{\text{i},\text{t}}$$ (2)

The interaction term ${\text{EPU}}_{\text{t}}\times {\text{ESG}}_{\text{i},\text{t}}\;$ allows us to assess whether ESG disclosure quality weakens (moderates) the negative relationship between policy uncertainty and firm valuation.

#### Extended model for economic consequences.

To assess whether EPU-induced reductions in firm value have additional economic consequences, we develop a mediation framework focusing on two channels: analyst attention and financial distress. The analysis proceeds in two stages.

First, we confirm the impact of EPU on firm value (as per Eq 1). Then, we examine whether TobinQ transmits the effect of EPU to two dependent outcomes: analyst coverage and financial distress. The models are specified as follows:


$${\text{TobinQ}}_{\text{i},\text{t}}=\gamma_{\text{0}}+\gamma_{\text{1}}{\text{EPU}}_{\text{t}}+\sum \gamma_{\text{m}}{\text{CVS}}_{\text{i},\text{t}}+\sum {\text{Ind}}_{\text{i}}+\sum {\text{Year}}_{\text{i}}+\varepsilon_{\text{i},\text{t}}$$ (3)

$${\text{Analyst}}_{\text{i},\text{t}}\text{ or }{\text{ZScore}}_{\text{i},\text{t}}=\delta_{\text{0}}+\delta_{\text{1}}{\text{TobinQ}}_{\text{i},\text{t}}+\sum \delta_{\text{m}}{\text{CVS}}_{\text{i},\text{t}}+\sum {\text{Ind}}_{\text{i}}+\sum {\text{Year}}_{\text{i}}+\varepsilon_{\text{i},\text{t}}$$ (4)

Here, ${\text{Analyst}}_{\text{i},\text{t}}\;$ is the natural logarithm of the number of analysts following firm i in year t plus one, reflecting capital market attention. Altman Z-score [33], where lower values indicate greater financial vulnerability ${\text{ZScore}}_{\text{i},\text{t}}$. represents the Altman Z-score [33], a widely used indicator of corporate financial distress. Lower Z-scores indicate higher likelihoods of bankruptcy. Bootstrap-based mediation tests are widely recommended for assessing indirect effects, particularly when normality and independence assumptions may be violated [34, 35]. The bootstrap confidence intervals yield results consistent with the Sobel test, reinforcing the robustness of the identified mediation effects.

If the effect of EPU on these outcome variables is significantly mediated by firm value (TobinQ), it supports the existence of an indirect consequence mechanism. We examine the mediation effects using a two-step fixed-effects regression framework and report Sobel test statistics as a supplementary diagnostic measure of indirect effects. Given the limitations of Sobel tests in panel-data settings, we further validate the mediation results using bootstrap-based confidence intervals with resampling at the firm level.

## 4 Empirical results and robustness checks

### Descriptive statistics and correlation analysis

Table 2 provides the descriptive statistics for the factors identified as significant. The average Tobin’s Q of 2.051 indicates that, generally, firms possess a market value exceeding their book value. The average ESG disclosure score for listed Chinese companies is 4.957, indicating a modest level of environmental, social, and governance transparency on a scale from 1 to 9. The broad distribution of EPU, with a standard deviation of 2.282 and an average of 4.957, indicates fluctuations in macroeconomic policy uncertainty during the study period.

**Table 2:** Descriptive statistic.

Variable	N	mean	sd	p50	min	max
ESG	34067	5.284	1.921	5.000	1.000	9.000
TobinQ	34607	2.051	1.379	1.623	0.795	17.73
EPU	34607	4.957	2.282	5.089	1.139	7.919
Lev	34607	0.421	0.204	0.411	0.040	0.938
Shb	34607	0.768	0.613	0.609	0.018	2.945
Roa	34607	0.036	0.070	0.036	−0.521	0.293
Age	34607	2.166	0.793	2.303	0.693	3.434
Ppe	34607	0.205	0.154	0.174	0.001	0.728
Cash	34607	0.191	0.336	0.119	−0.721	2.318
IIR	34607	0.421	0.246	0.432	0	0.917
Big4	34607	0.058	0.234	0	0	1

Table 3 displays among the key empirical study variables the Pearson correlation coefficients. With a correlation of −0.085 at 1% significant level 36 find that the value of a firm, indicated by Tobin’s Q, is negatively correlated with economic policy uncertainty (EPU). Tobin’s Q has a negative link with leverage (Lev), thereby confirming the theory that financially strong corporations generally have high market valuations even if it has a positive correlation with profitability (ROA) and operating cash flow (Cash). Generally low are correlations between control variables including business age (Age), fixed asset ratio (Ppe), and institutional ownership (IIR). There is not considerable multicollinearity risk for the regression investigations with all pairwise correlations much below the conventional multicollinearity threshold.

**Table 3:** Correlation matrix.

Variable	TobinQ	ESG	EPU	Lev	Shb	Roa	Age	Ppe	Cash	IIR	Big4
TobinQ	1										
ESG	0.062***	1									
EPU	−0.085***	−0.014*	1								
Lev	−0.239***	−0.091***	−0.026***	1							
Shb	0.052***	0.034***	0.102***	−0.097***	1						
Roa	0.137***	0.118***	−0.056***	−0.341***	−0.023***	1					
Age	−0.053***	−0.044***	0.016***	0.356***	−0.161***	−0.177***	1				
Ppe	−0.099***	−0.072***	−0.085***	0.081***	−0.078***	−0.046***	0.103***	1			
Cash	0.181***	0.096***	0.033***	−0.434***	0.027***	0.452***	−0.168***	0.064***	1		
IIR	−0.039***	0.083***	−0.073***	0.183***	−0.207***	0.120***	0.200***	0.136***	0.012**	1	
Big4	−0.059***	0.071***	0.014**	0.090***	−0.014**	0.038***	0.060***	0.029***	0.016***	0.239***	1

*, **, and *** indicate significance at the 10%, 5%, and 1% levels, respectively.

#### Baseline regression results.

Table 4 shows the baseline and moderated model panel regression results. First in Column (1) we regress Tobin’s Q on EPU (adding industry fixed effects but without any further variables). With a coefficient of EPU of −0.061 significant at the 1% level, hypothesis 2 more policy uncertainty is linked with less corporate value is confirmed. We incorporate the full collection of firm-level control factors in Column (2); EPU continues relatively negative of 0.066 is significant at the 1% level, so proving the result that EPU reduces market valuation after considering company fundamentals.

**Table 4:** Baseline and moderation regression results.

Variable	(1)	(2)	(3)
	TobinQ	TobinQ	TobinQ
EPU	−0.061***	−0.068***	−0.223***
	(−14.86)	(−16.04)	(−10.12)
EPU × ESG			0.039***
			(7.92)
ESG			−0.401***
			(−11.79)
Lev		−1.008***	−1.046***
		(−10.57)	(−11.32)
Shb		0.078***	0.075***
		(3.53)	(3.48)
Roa		0.691***	1.314***
		(2.91)	(5.68)
Age		0.135***	0.060***
		(6.81)	(2.92)
Ppe		−0.827***	−0.823***
		(−7.86)	(−8.07)
Cash		0.442***	0.456***
		(9.15)	(9.45)
Mgr		−0.630***	−0.493***
		(−5.96)	(−4.78)
IIR		−0.182	0.006
		(−1.27)	(0.08)
Big4		−0.246***	−0.150***
		(−4.38)	(−2.73)
_cons	2.523***	3.703***	4.528***
	(17.95)	(19.31)	(22.49)
Ind	YES	YES	YES
Year	NO	NO	NO
N	34607	34607	34607
Adjusted-R2	0.062	0.121	0.141

Note: 1) *, **, and *** indicate that the variables are significant at the 10%, 5%, and 1% levels, respectively; 2) the values in parentheses are the cluster-robust t-values.

Column (3) presents the interaction term among EPU and ESG disclosure quality. The interaction of EPU and ESG is significant and positive with a coefficient of 0.039 at the 1% level. This supports Hypothesis 3 since companies with higher ESG transparency show less severe value loss in response to policy uncertainty shocks. Stated otherwise, good ESG disclosure helps to offset the detrimental effect of EPU on corporate value.

Remarkably, at the 1% significant level the coefficient on ESG itself in this completely thorough model is negative coefficient of 0.399. This outcome implies that organizations with better ESG ratings usually have rather smaller contemporaneous Tobin’s Q. under constant EPU. From one point of view, as Flammer [15] emphasizes, the The negative coefficient on ESG disclosure quality reflects short-term adjustment and compliance costs associated with ESG implementation, particularly in emerging markets where ESG standards are still evolving. These costs may include increased reporting expenses, environmental investments, and organizational restructuring, which can exert downward pressure on firm valuation in the short run.

Importantly, this negative standalone effect does not contradict Hypothesis 1, which emphasizes the conditional value of ESG disclosure. Once economic policy uncertainty is taken into account, ESG disclosure exhibits a significant buffering effect through the interaction term, indicating that its primary economic role lies in mitigating downside risk rather than generating immediate valuation gains.

This result contrasts with our first expectation in Hypothesis 1, implying that while ESG disclosure is valuable as a risk-mitigating tool under uncertainty, its direct effect on valuation can be negative in the short run. We return to this subject in the debate since it emphasizes that, in the Chinese setting, ESG’s advantage may show itself basically as a moderating (insurance) effect rather than as an absolute performance booster.

Generally, the baseline and interaction regression results reveal that effective ESG disclosure can help to slightly mitigate the effect of EPU, which has a significant value-decreasing effect on companies. Leverage (Lev) walks in with a negative coefficient (businesses with greater debt have lower Q), ROA is positive (more profitable firms are valued higher), and other factors like ownership balance, firm age, etc., show intuitive effects and statistical relevance where appropriate. Usually speaking, the control variables have expected indications.

### Robustness checks

We run multiple robustness tests using different model parameters and variable definitions to make sure our main results are reliable. First, we substitute the price-to earnings (PE) ratio for Tobin’s Q to discuss corporate valuation. EPU remains particularly negatively associated with firm value as indicated in Columns (1) and (2) of Table 5; the interaction term between EPU and ESG disclosure quality stays positive and statistically significant. These findings verify that the moderating effect of ESG disclosure holds under several value criteria. We further re-estimate the baseline and moderation models with the inclusion of year fixed effects. Consistent with expectations, the explanatory power of the time-series EPU variable is reduced due to multicollinearity with year dummies. Importantly, however, the moderating effect of ESG disclosure quality on the EPU-firm valuation relationship remains statistically significant, confirming the robustness of our main conclusions.

**Table 5:** Robustness checks with alternative measures.

	(1)	(2)	(3)	(4)	(5)	(6)
Variable	Alternative Dependent Variable		Alternative Independent Variable		Alternative Moderating Variable	
	PE	PE	TobinQ	TobinQ	TobinQ	TobinQ (Year FE)
EPU	−7.495***	−27.402***			−0.611***	−0.183
	(−16.28)	(−10.83)			(−8.87)	(−1.12)
EPU × ESG		4.836***		0.027***		0.019**
		(8.89)		(12.11)		(2.41)
ESG		−42.793***		−0.333***		−0.218**
		(−11.33)		(−14.99)		(−2.36)
EPU1			−0.002***	−0.004***		
			(−17.17)	(−15.91)		
EPU1 × ESG				0.027***		
				(12.11)		
ESG1					−0.074***	
					(−11.73)	
EPU × ESG1					0.008***	
					(8.15)	
_cons	291.947***	455.125***	3.124***	4.406***	8.331***	2.015
	(13.89)	(16.82)	(20.19)	(24.52)	(17.45)	(1.31)
CVs	YES	YES	YES	YES	YES	YES
Ind	YES	YES	YES	YES	YES	YES
Year	NO	NO	NO	NO	NO	Yes
N	29814	29814	34607	34607	34607	34607
Adjusted-R2	0.144	0.161	0.126	0.147	0.140	0.192

Note: statistics in parentheses,*** and ** denote statistical significance at the 1% and 5% levels, respectively.

We then replace a news-based policy uncertainty measure created by derived from the frequency of uncertainty-related phrases in major Chinese newspapers for the original EPU index. The results in Columns (3) and (4) hold true with our baseline: the EPU coefficient is negative and significant while the interaction term of EPU and ESG disclosure stays positive and strong. Finally, we evaluate the sensitivity of our ESG measure by means of a continuous ESG score spanning 0–100 derived from the Huazheng Index. Emphasizing the reliability of our primary findings, the interaction between EPU and the alternative ESG score reported in Column (5) also produces a positive and significant coefficient.

We further validate our findings by subsample analysis and various variable definitions. We stratify the sample according to industry (manufacturing vs. non-manufacturing), geographic location (eastern vs. central/western China), and ownership type state-owned vs. private companies. EPU still has a considerably negative effect on firm value in all subsamples; the interaction between EPU and ESG remains positive and statistically significant. Fascinatingly, the buffering effect of ESG disclosure seems more noticeable in private companies perhaps because they depend more on voluntary transparency to create credibility and gain investor confidence. Likewise, companies in eastern areas more institutionally formed and driven by the market show a somewhat higher moderating influence of ESG. Still, the direction and importance of the results are the same everywhere. Comparisons at industry levels also show that, in times of policy-related uncertainty, both manufacturing and non-manufacturing companies gain from ESG disclosure. Variance inflation factor (VIF) diagnostics further indicate substantially elevated multicollinearity when both EPU and year fixed effects are included. Taken together, these results confirm that our main conclusions are not driven by specific modeling choices.

To ensure the statistical validity of the mediation results in a panel-data context, we additionally employ a bootstrap-based mediation test with resampling at the firm level. The bootstrap confidence intervals confirm that the indirect effects of economic policy uncertainty on analyst coverage and financial distress through firm value are statistically significant.

It is clear from these robustness testing that our major results are reliable and can be applied to a wide range of situations. Across many dependent and independent factors, scoring techniques, and business characteristics, the detrimental impact of economic policy uncertainty on firm valuation as well as the mitigating function of high-quality ESG disclosure holds. This consistency supports the theoretical assertion that ESG is a strategic asset improving company resilience in uncertain macroeconomic conditions as well as a compliance tool. Our findings demonstrate that the evidence supporting Hypotheses 2 and 3 is not dependent on particular modeling choices, therefore providing more assurance on the contribution of the study to the body of knowledge on ESG, policy uncertainty, and corporate value. As an additional diagnostic exercise, we further decompose ESG disclosure into its Environmental, Social, and Governance components to assess whether the moderating effect is driven by specific ESG dimensions.

### Subsample analyses by ownership, region, and industry

To further verify the generalizability and robustness of our results, we do subsample analyses by industry classification, geographic area, and company ownership. Table 6 displays the performance of privately held and state-owned enterprises. In both samples, economics policy uncertainty (EPU) has a quite negative coefficient; nonetheless, the interaction term between EPU and ESG disclosure quality is still rather positive. These results confirm that ESG disclosure reduces the connection in both environments whereas the negative effect of EPU on company value is constant across ownership forms. The moderating effect of ESG is more pronounced in private companies, and possibly because these companies depend more heavily on voluntary transparency techniques to build credibility and attract investment when there is no official relationship between them and the state.

**Table 6:** Results of state-owned and privately owned firms Analysis.

Variable	(1)	(2)	(3)	(4)
	State-Owned		Privately Owned Firms	
	TobinQ	TobinQ	TobinQ	TobinQ
EPU	−0.071***	−0.182***	−0.070***	−0.252***
	(−11.53)	(−6.36)	(−12.41)	(−8.25)
EPU × ESG		0.027***		0.046***
		(4.30)		(6.65)
ESG		−0.281***		−0.421***
		(−6.39)		(−9.13)
_cons	3.629***	4.591***	2.245***	4.157***
	(12.48)	(13.83)	(15.66)	(17.07)
CVs	YES	YES	YES	YES
Ind	YES	YES	YES	YES
Year	NO	NO	NO	NO
N	10797	10797	22540	22540
Adjusted-R2	0.193	0.210	0.101	0.119

Note: t statistics in parentheses,*** and ** denote statistical significance at the 1% and 5% levels, respectively.

### Mediation mechanism test

Table 7 shows the results of a subsample regression analysis comparing the locations of the headquarters of enterprises in eastern and central-western China. With reductions of −0.067 and −0.218 in the East and −0.070 and −0.237 in the Central-West, both at a 1% significance level, economic policy uncertainty (EPU) greatly lowers company value in both regions. In relation with baseline models, a combination of values of 0.037 in the East and 0.042 in the Central-West both at 1% significant level, the interaction term of EPU and ESG disclosure shows a positive and statistically significant moderating impact of ESG disclosure.

**Table 7:** Results of regional subsample analysis.

Variable	(1)	(2)	(3)	(4)
	Eastern Regions		Central-Western Regions	
	TobinQ	TobinQ	TobinQ	TobinQ
EPU	−0.067***	−0.218***	−0.070***	−0.237***
	(−13.41)	(−8.62)	(−8.45)	(−5.31)
EPU × ESG		0.037***		0.042***
		(6.70)		(4.26)
ESG		−0.373***		−0.453***
		(−9.89)		(−6.45)
_cons	2.592***	4.324***	2.837***	4.920***
	(22.56)	(21.50)	(10.45)	(12.33)
CVs	YES	YES	YES	YES
Ind	YES	YES	YES	YES
Year	NO	NO	NO	NO
N	24862	24862	9741	9714
r2_a	0.125	0.146	0.116	0.148

Note: t statistics in parentheses,*** and ** denote statistical significance at the 1% and 5% levels, respectively.

Since the eastern regions, where ESG has a rather larger influence, have more developed institutional infrastructure, general tendencies remain constant throughout all provinces. The results show that the quality of ESG always reduces the negative value effects of policy uncertainty independent of geographical differences in market maturity or institutional development.

Table 8 presents industry-based regression results split between manufacturing and non-manufacturing sectors. Economic policy uncertainty (EPU) significantly decreases company value (Tobin’s Q) in both sectors; coefficients for economic policy uncertainty vary from −0.054 to −0.240, all statistically significant at the 1% level. The interaction term EPU of ESG disclosure is positive and statistically significant in both subsamples with values of 0.043 in manufacturing and 0.034 in non-manufacturing at the 1% significance level, therefore displaying the constant moderating effect of ESG disclosure quality.

**Table 8:** Industry subsample analysis: Manufacturing vs. Non-Manufacturing firms.

	(1)	(2)	(3)	(4)
Variable	Manufacturing		Non-Manufacturing	
	TobinQ	TobinQ	TobinQ	TobinQ
EPU	−0.054***	−0.225***	−0.100***	−0.240***
	(−10.51)	(−8.36)	(−12.87)	(−5.95)
EPU × ESG		0.043***		0.034***
		(7.10)		(3.99)
ESG		−0.406***		−0.396***
		(−9.99)		(−6.68)
_cons	2.358***	4.247***	2.972***	4.840***
	(27.75)	(21.45)	(16.97)	(15.36)
CVs	YES	YES	YES	YES
Ind	YES	YES	YES	YES
Year	NO	NO	NO	NO
N	23132	23132	11471	11471
Adjusted-R2	0.081	0.102	0.185	0.212

Note: t statistics in parentheses,*** and ** denote statistical significance at the 1% and 5% levels, respectively.

The findings validate that the reducing influence of ESG disclosure seems outside of a specific industrial setting. Strong ESG regulations serve to lower the negative valuation consequences of macroeconomic uncertainty, therefore influencing both capital-intensive industrial and service-oriented industries.

### Extended analysis: Analyst coverage and financial distress

The purpose of this study is to explore more within Hypothesis 4 by exploring how economic policy uncertainty (EPU) affects company valuation in the long run. We emphasize on how EPU affects financial distress and analyst coverage. The mediator analysis is presented in Table 9. Based on Tobin’s Q, baseline results reveal that firm value is much reduced by a rise in EPU. In the second stage we regress analyst coverage and financial distress, as revealed by the Altman Z-score, on Tobin’s Q. While controlling for EPU and firm-specific factors.

**Table 9:** The mediation analysis results of analyst coverage and financial distress.

Variable	(1)	(2)	(3)	(4)
	TobinQ	Analyst	TobinQ	Z-score
EPU	−0.030***		−0.044***	
	(−3.54)		(−6.41)	
TobinQ		0.073***		3.310***
		(11.08)		(14.35)
_cons	−0.172	1.050	−4.729	2.832**
	(−0.01)	(8.12)	(−0.48)	(2.45)
CVs	YES	YES	YES	YES
Ind	YES	YES	YES	YES
Year	NO	NO	NO	NO
N	22,994	22,994	34,604	34,604
Adjusted-R2	0.333	0.205	0.312	0.215
SobelTest	(Z = −3.52, P = 0.00)		(Z = --6.41, P = 0.00)	

*, **, and *** indicate significance at the 10%, 5%, and 1% levels, respectively.

The favorable correlation between Tobin’s Q and analyst coverage reveals supports for Hypothesis 4(a). As economic policy uncertainty (EPU) leads coverage of companies with higher Tobin’s Q values to reduce, market transparency declines and analysts pay greater attention to companies with lower q values. Tobin’s Q indicates a positive correlation with the Z-score, thereby supporting Hypothesis 4(b): a lower company value is related with higher financial problems. Including Tobin’s Q into the study yields a substantially less direct impact of EPU on both outcomes, implying that EPU largely affects analyst coverage and financial health via means of its effect on company value.

Sobel tests show that these indirect effects are relevant as shown by Z-statistics of −3.52 for analyst coverage and −6.41 for financial difficulty, both significant at the 1% level. The results highlight the more general systematic risks associated with policy uncertainty, which not only lower market value but also affect the financial stability and visibility of businesses. As a result of ESG disclosure, the valuation impact of EPU is significantly reduced, leading to a more robust informational and financial context.

## 5 Discussion and conclusion

### Conclusion

This study examines how ESG disclosure quality shapes the relationship between economic policy uncertainty and firm valuation in an emerging-market context. Drawing on a large panel of Chinese A-share listed companies, the analysis demonstrates that ESG disclosure plays an important role in moderating the adverse valuation effects associated with heightened policy uncertainty.

### Practical implication and future research direction

From a theoretical perspective, the findings extend existing research by integrating the economic policy uncertainty literature with studies on ESG disclosure. Rather than viewing ESG disclosure solely as a long-term value-enhancing practice, the results highlight its function as a mechanism that helps firms manage uncertainty and stabilize investor expectations during periods of policy turbulence. This perspective underscores the state-dependent value of ESG disclosure and enriches theoretical discussions on information asymmetry and signaling under uncertainty.

The results also offer meaningful implications for practice and policy. For corporate managers, credible and consistent ESG disclosure should be regarded as part of a broader strategy for risk management and investor communication, particularly in uncertain policy environments. For investors, ESG information provides a useful signal for assessing firm resilience when macroeconomic conditions become volatile. For regulators and policymakers, the findings support efforts to promote standardized and credible ESG disclosure frameworks as a means of enhancing market transparency and mitigating uncertainty-driven distortions in firm valuation.

Despite these robustness checks, the possibility of residual endogeneity cannot be fully ruled out, as firms with higher valuation may have greater incentives or resources to invest in ESG disclosure. Future research could exploit exogenous policy shocks or instrumental-variable strategies to further strengthen causal identification.

Several limitations suggest directions for future research. Further work could examine whether third-party ESG assurance strengthens the credibility of disclosure, explore how differences among ESG rating agencies affect investor responses under uncertainty, or conduct cross-country analyses to assess how institutional environments shape the uncertainty-buffering role of ESG disclosure. Such research would deepen understanding of how sustainability-related information contributes to firm resilience in challenging economic conditions.

### Discussion

Firm valuation in China is highly sensitive to economic policy uncertainty, reflecting the prominent role of government intervention and frequent regulatory adjustments in shaping market expectations. When policy signals become less predictable, investors face greater difficulty in forming expectations about future cash flows and regulatory conditions, which increases perceived risk and depresses firm valuation. This pattern is consistent with the broader literature showing that policy-related uncertainty weakens investor confidence, raises risk premia, and discourages long-term investment, particularly in institutional environments where firms are closely tied to policy direction.

Against this backdrop, ESG disclosure functions as an informational and governance mechanism rather than merely a compliance-related activity. Firms with higher-quality ESG disclosure are better able to communicate long-term strategic orientation, governance stability, and commitment to key stakeholders when policy uncertainty intensifies. By reducing ambiguity surrounding managerial intentions and organizational resilience, transparent ESG disclosure helps stabilize investor perceptions at times when external policy signals are volatile. As a result, the valuation relevance of ESG disclosure appears to be state-dependent, becoming more pronounced precisely when macroeconomic uncertainty is elevated.

Heterogeneity across ownership structures, regions, and industries further highlights the role of institutional context. The stronger buffering effect of ESG disclosure among private firms and firms located in more market-oriented regions suggests that voluntary transparency plays a more meaningful role when external governance mechanisms and investor scrutiny are stronger. In contrast, firms operating under greater state influence may rely less on disclosure to signal resilience, as alternative forms of implicit support or governance arrangements shape investor expectations.

This context-dependent valuation pattern aligns with prior evidence indicating that ESG-related activities are not uniformly priced by the market [36]. Fatemi et al. (2018) [37] show that ESG performance alone does not necessarily enhance firm value and that valuation effects critically depend on the quality and credibility of disclosure. Extending this insight to a macroeconomic uncertainty setting, our findings suggest that ESG disclosure becomes particularly value-relevant when economic policy uncertainty intensifies, reinforcing its role as an informational buffer rather than a constant driver of firm value. In periods of heightened uncertainty, investors appear to place greater weight on credible disclosure as a signal of governance quality and long-term resilience.

The mediation analysis further reveals that the effects of policy uncertainty extend beyond immediate valuation changes. Declines in firm value associated with heightened uncertainty are accompanied by reduced analyst coverage and increased financial vulnerability, indicating that valuation shocks propagate through the information environment and firms’ financial conditions. As market value deteriorates, information asymmetries widen and external monitoring weakens, which in turn exacerbates financial stress. These dynamics point to a self-reinforcing mechanism through which economic policy uncertainty undermines not only firm valuation but also information transparency and financial stability.

## Supporting information

S1 Data R1 anonymized data. (XLSX)
